# Biomarkers of Oxidative Stress and Inflammation in Chronic Kidney Disease: A Cross-Sectional Study of Vitamin C and Pentraxin 3 Dynamics During Hemodialysis

**DOI:** 10.7759/cureus.83480

**Published:** 2025-05-04

**Authors:** Vignesh Babu K, Bharath Rajh

**Affiliations:** 1 Biochemistry, Arunai Medical College and Hospital, Tiruvannamalai, IND; 2 Community Medicine, Velammal Medical College Hospital & Research Institute, Madurai, IND

**Keywords:** chronic kidney disease, hemodialysis, inflammation, oxidative stress, pentraxin 3, vitamin c

## Abstract

Introduction: Chronic kidney disease (CKD) is a condition where there is a progressive decline in kidney function, which is characterized by an estimated glomerular filtration rate of less than 60 mL/min/1.73 m². Vitamin C is a strong antioxidant, and pentraxin 3 (PTX3) is an early reliable sign of vascular inflammation and cardiovascular risk. However, it is still not clear how these two markers interact with each other during hemodialysis. This study aimed to correlate vitamin C and pentraxin-3 levels pre- and post-dialysis among CKD patients.

Methods: A prospective observational study was conducted at Dhanalakshmi Srinivasan Medical College and Hospital, involving 60 CKD patients aged more than 18 years who were on maintenance hemodialysis. After obtaining written informed consent, we collected the participants' demographic information, clinical data, and biochemical parameters from their medical records. We used high-performance liquid chromatography (HPLC) and enzyme-linked immunosorbent assay (ELISA) to check the levels of vitamin C and PTX3 in the blood sample taken 30 minutes before and after dialysis. Vitamin C levels were classified as deficient, low, or normal, and pentraxin 3 values were categorized as normal, mildly elevated, moderately elevated, and severely elevated. A paired t-test was used to assess pre- and post-dialysis variations among the blood biochemical parameters. Pearson's correlation analysis was done to determine the correlation between vitamin C and pentraxin 3 in hemodialysis patients.

Results: The study population had a mean age of 52.88 ± 9.55 years, with 51 (85%) males. Vitamin C levels dropped significantly after dialysis from 0.24 ± 0.092 mg/dL to 0.19 ± 0.085 mg/dL (p = 0.001), but PTX3 levels increased from 4.64 ± 1.299 ng/mL to 6.63 ± 1.259 ng/mL (p = 0.001) after dialysis. A total of 40 (66.67%) study participants were found to have low vitamin C levels; vitamin C deficiency was present in 19 (31.67%) participants in a pre-dialysis state, and their condition worsened further post-dialysis. A total of 31 (51.67%) participants had severely elevated PTX 3 levels, which further increased post-dialysis. PTX3 levels were consistently higher in hypertensive and diabetic patients, highlighting the inflammatory burden in these populations. There exists a weak, nonsignificant negative correlation between vitamin C and PTX 3 levels both pre- (r = -0.137, p = 0.781) and post-dialysis (r = -0.141, p* *= 0.757).

Conclusion: Although this observational study cannot establish causality, it indicates that hemodialysis is associated with reduced antioxidant levels and increased inflammatory markers. These findings suggest a possible link between oxidative stress and inflammation in CKD patients. Further studies are needed to evaluate the therapeutic role of antioxidant supplementation in mitigating cardiovascular risks.

## Introduction

Chronic kidney disease (CKD) is defined as a progressive condition where the kidneys lose their ability to do their primary function effectively. It is characterized by the presence of kidney damage or an estimated glomerular filtration rate (eGFR) of less than 60 mL/min/1.73 m^2^, lasting for a minimum of three months or longer [1]. In India, the prevalence of CKD varies, with estimates ranging from 8.7% in Tamil Nadu (Madras Medical College, 2023) to 13.24% nationally (Talukdar et al., 2023), with higher rates in rural areas [2, 3]. Additionally, a higher incidence of CKD was observed in rural areas of India, as noted by Anupama et al. in 2014 [4]. The two major causes of nephropathy progressing to CKD were diabetes mellitus and systemic hypertension, which harm the kidneys through various mechanisms [5]. The National Kidney Foundation classifies CKD into five stages based on glomerular filtration rate (GFR). Stage 1 (GFR ≥ 90) is characterized by normal kidney function without any damage. Stage 2 (GFR 60-89) indicates a mild reduction in kidney function. Stage 3 (GFR 30-59) represents moderate impairment, while Stage 4 (GFR 15-29) reflects severe kidney damage. Stage 5 (GFR <15) indicates end-stage renal disease, where kidney function is severely compromised and dialysis may be required [1, 6].

Vitamin C is also known as ascorbic acid; it is necessary for antioxidant defense, iron absorption, and tissue regeneration. The recommended daily allowance for vitamin C was fixed at 90 mg/day for men and 75 mg/day for women, as fixed by the National Institute of Health [7]. Additionally, it has antioxidant and anti-inflammatory actions, which help especially in patients suffering from CKD [8]. It reduces oxidative stress by scavenging reactive oxygen species (ROS) and reactive nitrogen species (RNS). Oxidative stress levels are significantly higher in patients with CKD because of the buildup of uremic toxins, persistent inflammation, and repeated dialysis exposure. This heightened oxidative environment accelerates vascular damage and cardiovascular morbidity among CKD patients [8]. CKD patients undergoing hemodialysis frequently exhibit vitamin C deficiency, necessitating prophylactic supplementation to support antioxidant activity [9]. Studies show that increased blood vitamin C levels may lower the progression of CKD in some patients, whereas it is evident that lower antioxidant levels are associated with a higher inflammatory process [10].

Pentraxin 3 (PTX3) is a pattern recognition receptor that is mainly involved in innate immunity and systemic inflammation. Many different types of cells, including endothelial cells and macrophages, made it in response to inflammatory substances like interleukin-1, tumor necrosis factor-α, and lipopolysaccharides (LPS) [11]. The normal value of PTX3 is less than 2 ng/ml. Heart failure, unstable angina/non-ST-elevation myocardial infarction (UAP/NSTEMI) [12, 13], and acute myocardial infarction (AMI) [14], as well as higher PTX3 levels, are all linked to CKD and hemodialysis patients. Increased PTX3 not only predicts severe cardiac events but is also linked to worse long-term outcomes in patients with heart failure [15]. Patients on hemodialysis who had end-stage renal disease had significantly higher PTX3 levels, which was linked to a lower five-year survival rate [16]. PTX3 has been a more reliable indicator for predicting all-cause mortality, cardiovascular mortality, and other adverse outcomes than CRP/hs-CRP in CKD patients [16]. On the other hand, PTX3 helps to predict the progression of diabetic nephropathy by distinguishing microalbuminuria and macroalbuminuria [12, 13]. As per the literature, the PTX3 levels rise significantly after hemodialysis, implicating that the procedure itself induces higher inflammation, further reinforcing that PTX3 is a sensitive biochemical marker for dialysis-related inflammatory processes and cardiovascular risk among CKD patients. Factors such as diabetes, hypertension, malnutrition, and dialysis duration may influence these biomarkers, potentially confounding observed associations.

CKD is a progressive illness associated with high morbidity and mortality. A multicenter Asian study, including patients from India, was conducted by Wong et al., which reported a five-year survival rate of approximately 36% among hemodialysis patients, reflecting poorer outcomes compared to high-income countries [17]. This underscores the urgent need to address systemic inflammation and oxidative stress, which are key contributors to mortality in this population. While PTX3 and vitamin C have been independently linked to CKD outcomes, their interaction in hemodialysis patients, particularly in southern Tamil Nadu, remains unexplored. Vitamin C’s antioxidant properties may potentially modulate PTX3-mediated inflammation, warranting investigation. Therefore, this study aims to assess the relationship between vitamin C and PTX3 in CKD patients undergoing hemodialysis.

Objectives

This study aims (1) to evaluate and compare serum vitamin C and pentraxin 3 (PTX3) levels before and after hemodialysis in patients with CKD and (2) to assess the correlation between serum vitamin C levels and pentraxin 3 levels in CKD patients undergoing hemodialysis and explore the influence of comorbid conditions such as diabetes mellitus and hypertension.

## Materials and methods

A hospital-based observational study was conducted in the Department of Biochemistry, Dhanalakshmi Srinivasan Medical College and Hospital, Perambalur, India. The duration of the study period was six months from 1st April 2016 to 30th September 2016, a retrospective analysis of prospectively collected data. The sample size was calculated based on the prevalence of chronic renal disease at 8.7%, in Tamil Nadu (2015), with a 95% confidence interval and absolute precision at 7% [18]. A minimum of 60 hemodialysis patients was necessary to conduct the study. The study population included adult patients above the age of 18 with CKD who had been receiving hemodialysis for a minimum of three months at the nephrology ward. On the other hand, patients with active infections, recent hospitalizations, or ongoing vitamin C supplementation were excluded from the study.

Institutional ethical committee approval was obtained before the initiation of the study (IEC No.: IECHS/DSMCH/018/Version_1). After obtaining written informed consent, a semi-structured questionnaire was used to collect patient data from medical records, including blood pressure readings, comorbidities, demographics, and blood reports of serum urea, creatinine, and electrolytes. Serum vitamin C and PTX3 levels were measured using high-performance liquid chromatography (HPLC) and enzyme-linked immunosorbent assay (ELISA), respectively, in blood samples collected 30 minutes pre- and post-dialysis to capture immediate dialysis effects [14]. The researcher covered the expenses of the blood investigation (vitamin C and PTX3), ensuring that study participants did not incur any costs. We entered the collected data into Microsoft Excel (Microsoft Corp., Redmond, Washington), and data analysis was done by R programming software version 4.4.3. The demographic details were expressed by frequency and percentage, while the biochemical parameters were expressed in terms of mean and standard deviation for both pre- and post-dialysis. Based on laboratory values, vitamin C levels were categorized into three groups: deficient (<0.20 mg/dL), low (0.20-0.39 mg/dL), and normal (>0.40 mg/dL) [19, 20]. PTX3 values less than 2 were considered normal, above which was categorized as mildly (2.0-3.5 ng/ml), moderately (3.51-4.5 ng/ml), and severely elevated (>4.5 ng/ml) based on the quartiles. A paired t-test was conducted to assess the degree and statistical significance of the correlations. Pearson's correlation test was applied to determine the correlation between vitamin C and pentraxin 3 levels in hemodialysis patients.

## Results

This study enrolled 60 participants with CKD undergoing hemodialysis. The study participants' mean age ranged from 52.88 to 9.551 years (Table 1). The majority of them (66.7%) were in the 46-60 age category, with male predominance (85%). Our study revealed that 66.7% of patients had hypertension, and 35% had diabetes mellitus (Table 1). Of these, 15 CKD patients (25%) had manifested both diabetes mellitus and hypertension.

**Table 1:** Sociodemographic details of the study participants

S. No.	Variables	Categories	Frequency (n = 60)	Percentage
1.	Age (years)	18-30	1	1.7%
		31-45	9	15.0%
		46-60	40	66.7%
		≥61	10	16.6%
2.	Sex	Male	51	85.0%
		Female	9	15.0%
3.	Diabetes mellitus	Yes	21	35.0%
		No	39	65.0%
4.	Hypertension	Yes	40	66.7%
		No	20	33.3%

Table 2 displays the biochemical parameter values pre- and post-dialysis. Serum urea values significantly dropped from 103.51 ± 36.64 mg/dl to 64.81 ± 20.24 mg/dl post-dialysis (p = 0.001); similarly, serum creatinine levels were 9.39 ± 2.87 mg/dl before dialysis and dropped to 4.95 ± 1.57 mg/dl after dialysis (p = 0.001). Serum potassium levels significantly decreased from 5.34 ± 0.666 mEq/L to 3.94 ± 0.322 mEq/L (p = 0.001), while serum sodium levels declined from 141.55 ± 3.105 mEq/L to 136.45 ± 2.110 mEq/L (p = 0.001), which was statistically significant. There was a minor decrease in chloride levels from 103.83 ± 4.741 mEq/L to 102.06 ± 4.356 mEq/L (p = 0.001). In terms of vitamin C, there was a significant drop in level post-hemodialysis, which went from 0.24 ± 0.092 mg/dl to 0.19 ± 0.085 mg/dl (p = 0.001). In contrast, pentraxin 3 (PTX3) levels went up sharply after hemodialysis, from 4.64 ± 1.299 ng/ml to 6.63 ± 1.259 ng/ml (p = 0.001) (Table 2), which is a very important change.

**Table 2:** Paired t-test analysis of outcome variables with respect to hemodialysis (HD)

S. No.	Outcome variables	Reference value	Categories	Mean	SD	t-value	p-value
1.	Urea (mg/dL)	7-20 mg/dl	Before HD	103.51	36.643	16.880	0.001
			After HD	64.81	20.242		
2.	Creatinine (mg/dL)	0.7-1.3 mg/dl	Before HD	9.39	2.871	22.849	0.001
			After HD	4.95	1.572		
3.	Sodium (mEq/L)	135-145 mEq/L	Before HD	141.55	3.105	17.595	0.001
			After HD	136.45	2.110		
4.	Potassium (mEq/L)	3.5-5.0 mEq/L	Before HD	5.34	0.666	20.649	0.001
			After HD	3.94	0.322		
5.	Chloride (mEq/L)	98-107 mEq/L	Before HD	103.83	4.741	12.679	0.001
			After HD	102.06	4.356		
6.	Vitamin C (mg/dL)	0.4-2.0 mg/dl	Before HD	0.24	0.092	17.996	0.001
			After HD	0.19	0.085		
7.	Pentraxin 3 (ng/ml)	<2.0 ng/ml	Before HD	4.64	1.299	-28.589	0.001
			After HD	6.63	1.259		

**Figure 1:**
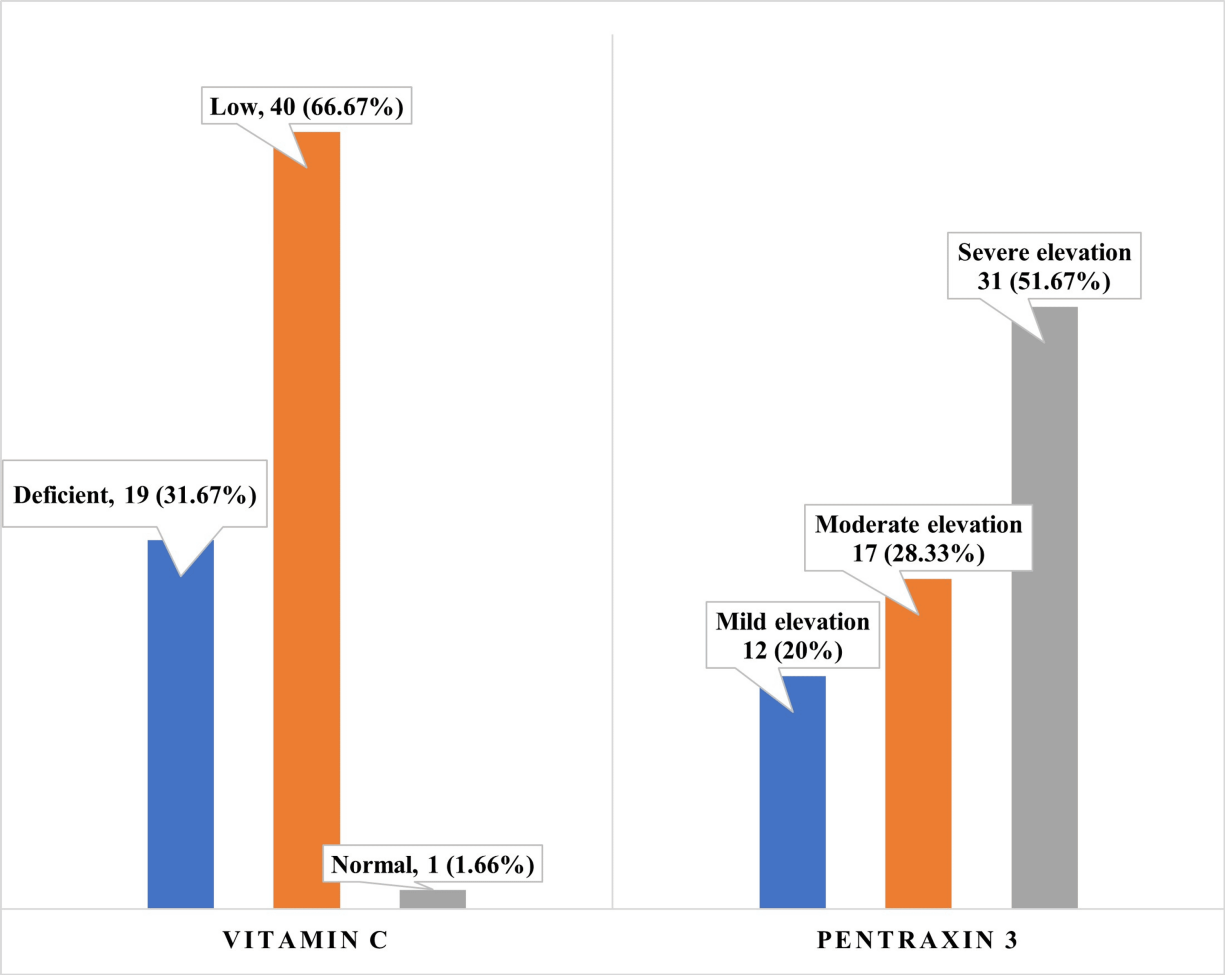
Categorization of vitamin C and pentraxin 3 based on the pre-dialysis values

Based on test results, vitamin C was divided into three groups: deficient (<0.20 mg/dl), low (0.20-0.39 mg/dl), and normal (>0.40 mg/dl). Before dialysis, the majority of study participants had low vitamin C levels; 40 (66.67%) participants fell into the low vitamin C level group, and 19 (31.67%) participants had vitamin C deficiency (Figure 1). Only one participant (1.66%) had adequate vitamin C levels, i.e., more than 0.4 mg/dl, which indicates that all CKD patients are more prone to vitamin C deficiency. They further worsen post-dialysis. All 60 participants had elevated PTX3, indicating an inflammatory state in CKD patients. The majority of study participants exhibited a severe elevation of PTX3 (>4.5 ng/ml) with 31 (51.67%) CKD patients, followed by moderate elevation in PTX3 values seen in 17 (28.33%) participants, and the rest (12; 20%) fell into the mild elevation category. These values significantly increased post-dialysis as all the participants had values of more than 4.5 ng/ml and a mean value of PTX3 post-dialysis was found to be 6.63 ± 1.259 ng/ml.

A paired t-test compared pre- and post-dialysis vitamin C and PTX3 levels, stratified by comorbidities (diabetes mellitus and hypertension) (Table 3). No adjustments were made for potential confounders such as age or dialysis duration due to the small sample size. There is a significant drop in vitamin C levels among both diabetic and non-diabetic populations (p = 0.001), signifying that dialysis causes a similar decline in the diabetic and non-diabetic populations. In both groups, there was an increase in PTX3 levels, which was statistically significant (p = 0.001), suggesting an increase in inflammation following hemodialysis. Patients with and without hypertension showed similar results, with both markers demonstrating significant changes (p = 0.001). PTX3 levels were consistently greater in hypertensive individuals before and after hemodialysis, indicating increased inflammation.

**Table 3:** Subgroup analysis to determine the impact of diabetes mellitus and hypertension on vitamin C and pentraxin 3 in CKD patients undergoing hemodialysis Paired t-test was used. CKD: Chronic kidney disease.

S. No.	Outcome variables	Categories	Vitamin C (mg/dl)					Pentraxin 3 (ng/ml)		
			Pre-HD Mean ± SD	Post-HD Mean ± SD	t-value	P-value	Pre-HD Mean ± SD	Post-HD Mean ± SD	t-value	P-value
1.	Diabetes mellitus	Yes	0.25 ± 0.11	0.20 ± 0.10	9.35	0.001	4.67 ± 1.19	6.68 ± 1.23	-15.143	0.001
		No	0.24 ± 0.08	0.18 ± 0.07	15.508	0.001	4.61 ± 1.36	6.60 ± 1.28	-24.416	0.001
2.	Hypertension	Yes	0.25 ± 0.94	0.19 ± 0.08	15.408	0.001	4.85 ± 1.27	6.87 ± 1.21	-23.186	0.001
		No	0.23 ± 0.08	0.18 ± 0.08	9.299	0.001	4.21 ± 1.27	6.14 ± 1.22	-16.570	0.001

Pearson’s correlation test was used to assess the association between vitamin C and PTX3 levels, after confirming the normal distribution of data via the Shapiro-Wilk test. Nonparametric alternatives (e.g., Spearman’s correlation) were considered if assumptions were violated. The results revealed a weak, nonsignificant negative correlation between serum vitamin C and PTX3 levels in hemodialysis patients, with the Pearson correlation coefficient of -0.137 (p = 0.781) predialysis and -0.141 (p = 0.757) post-dialysis. Even though it is not statistically significant, the negative correlation implies that there is a potential inverse relationship, where lower antioxidant levels might be associated with higher inflammation.

## Discussion

This study investigated the association between vitamin C and PTX3 levels in CKD patients undergoing hemodialysis. Vitamin C’s antioxidant and anti-inflammatory properties may theoretically counteract PTX3-mediated vascular inflammation, a sensitive marker of cardiovascular risk [21]. However, the weak, nonsignificant correlation suggests complex interactions influenced by dialysis, comorbidities, or nutritional factors. Given the increased oxidative stress and inflammatory load in hemodialysis patients, it is imperative to comprehend how these indicators interact.

There were 60 participants in all, and the majority (66.7%) were between the ages of 46 and 60 with male preponderance. Similarly, the majority of participants in research by Shruthi et al. on CKD patients receiving hemodialysis were between the ages of 41 and 50 (62%), and 86% of them were male [22]. Our study's mean blood pressure was 147.25 ± 24.41 mmHg, which is consistent with previous research where most individuals had readings between 140 and 160 mmHg [22].

Changes in biochemical parameters pre- and post-hemodialysis

Following hemodialysis, there were notable alterations in blood biochemical indicators such as urea, creatinine, sodium, potassium, and chloride. The levels of sodium in the serum fell from 141.55 to 136.45 mEq/L, potassium from 5.34 to 3.94 mEq/L, and chloride from 103.83 to 102.06 mEq/L. Certain results are consistent with those of Correa et al., who found that certain metabolic markers significantly decreased after dialysis [23]. In a similar vein, Soumya et al. found that potassium levels significantly decreased (from 5.1 to 3.3 mEq/L), whereas sodium and chloride levels barely changed [24].

Hemodialysis involves filtering blood via a dialyzer, often known as an artificial kidney, to eliminate excess waste products including creatinine and urea. Serum creatinine levels in our research fell from 9.39 ± 2.87 mg/dl to 4.95 ± 1.57 mg/dl, and serum urea levels declined considerably from 103.51 ± 36.64 mg/dl to 64.81 ± 20.24 mg/dl. This decrease was in line with earlier research and was statistically significant. For example, after dialysis, urea levels decreased from 108.60 ± 26.70 mg/dl to 48.54 ± 17.48 mg/dl, and creatinine decreased from 10.07 ± 3.82 mg/dl to 4.91 ± 1.98 mg/dl, according to Bhuvaneshwari et al. [25]. Likewise, Gulavani et al. documented a significant decrease in creatinine and urea levels following hemodialysis [26]. These findings support hemodialysis’s ability to remove nitrogenous waste and address electrolyte imbalances.

Vitamin C and PTX3 dynamics

In this study, vitamin C levels were measured both before and after hemodialysis. Just one (1.66%) subject had normal vitamin C levels based on predialysis measurements. Vitamin C levels consistently decreased after hemodialysis, making patients even more vulnerable to oxidative stress. Zhang et al.'s research, which found that only 25.7% of patients with CKD had normal vitamin C levels, is consistent with this. They also found that lower vitamin C concentrations were linked to higher C-reactive protein, which indicates inflammation [27]. Post-dialysis vitamin C deficiency underscores the potential role of supplementation to mitigate oxidative stress in CKD patients. However, interventional studies are needed to confirm whether restoring vitamin C levels reduces PTX3 or improves clinical outcomes. By emphasizing the connection between oxidative stress and inflammation in CKD, our findings highlight the potential benefits of vitamin C administration.

Post-dialysis, PTX3 levels significantly increased, confirming hemodialysis as an inflammatory trigger. This elevation, particularly in hypertensive and diabetic patients, suggests heightened cardiovascular risk, warranting exploration of anti-inflammatory strategies. Researchers have established that elevated PTX3 levels indicate cardiovascular risk and vascular inflammation. Xu et al. showed that PTX3 levels increased significantly after dialysis (2.18 vs. 1.87 ng/mL), and they were higher in people who were on hemodialysis than those in healthy controls [28]. A study by Li et al. [16] found that CKD patients with higher PTX3 levels had a higher risk of dying from heart disease (HR, 1.98; 95% CI, 1.28-3.05), an infection (HR, 5.26; 95% CI, 1.60-17.31), and any cause (HR, 1.92; 95% CI, 1.44-2.56).

Limitations

This study has several limitations. The small sample size (n = 60) and single-center recruitment reduce generalizability. Unmeasured confounders, such as dialysis membrane type, duration, or nutritional status, may have influenced vitamin C and PTX3 levels. The cross-sectional design limits analysis to short-term dialysis effects, precluding evaluation of long-term outcomes such as cardiovascular events or mortality. The study did not measure other relevant markers, such as C-reactive protein or malondialdehyde, which could provide a more comprehensive view of oxidative stress and inflammation in CKD. Including such parameters could have provided a more comprehensive understanding of the inflammatory cascade in CKD.

Recommendations

From this study's findings, it is evident that the vitamin C levels decrease and PTX3 levels increase post-dialysis. This observation signifies the interplay between oxidative stress and inflammatory processes among CKD patients. Regular monitoring of key biochemical parameters, including vitamin C, PTX3, and electrolytes, is advised for CKD patients on hemodialysis. Vitamin C supplementation may be considered for those with confirmed deficiency, pending validation from randomized controlled trials.

## Conclusions

This study highlights the interplay between oxidative stress and inflammation in CKD patients undergoing hemodialysis, with reduced vitamin C levels and elevated PTX3 post-dialysis signaling heightened cardiovascular risk. Low vitamin C levels were observed in 40 patients (66.67%), and vitamin C deficiency was observed in 19 CKD patients (31.67%). Vitamin C levels worsened further following hemodialysis, making them more vulnerable to oxidative damage. Most of the people in the study had PTX3 levels that were very high in 31 participants (51.67%), and these levels increased sharply after hemodynamic dialysis, which supports the idea that the procedure caused inflammation. Hemodialysis exacerbates systemic inflammation and depletes antioxidants, potentially contributing to cardiovascular risk, as indicated by elevated PTX3 levels. Further studies are needed to confirm these associations and evaluate preventive strategies.
